# Microbiota responses to feed particle size, calcium concentration, and phytase supplementation in broiler chickens

**DOI:** 10.1016/j.psj.2025.106209

**Published:** 2025-12-06

**Authors:** Ismael Rubio-Cervantes, Stephanie Wolfrum, Wolfgang Siegert, Markus Rodehutscord, Amélia Camarinha-Silva

**Affiliations:** a Institute of Animal Science, University of Hohenheim, 70599, Stuttgart, Germany; b Department of Animal Sciences, University of Göttingen, 37077 Göttingen, Germany

**Keywords:** Microbiota, Particle size, Phytase, Calcium

## Abstract

This research aimed to investigate the modulation of broiler-chickens gut microbiota by dietary particle size (PS), exogenous phytase, and calcium (Ca) concentration. Eight experimental diets varied in PS (fine 222 µm (PF) and coarse 309 µm (PC)), Ca concentration (4.9 and 7.2 g/kg), and exogenous phytase (0 and 1000 FTU/kg). A total of 560 Ross 308 broiler chickens were allocated to 56 metabolism units at 7 days of age and randomly assigned to each diet (7 replicates per treatment). On days 22 and 23, the birds were slaughtered, and the digesta from the gizzard, ileum, and ceca were sampled and pooled on a metabolism unit basis. DNA extraction was followed by 16S rRNA gene amplicon sequencing.

Thirteen amplicon sequence variants (ASV) were present across the gizzard, ileum, and ceca, most of which were assigned to *Limosilactobacillus* and represented a substantial share of the total relative abundance in each section, 86 % in the gizzard, 88 % in the ileum, and 30 % in the ceca. Six of these *L. reuteri* ASVs were significantly enriched by coarse particle feeding, suggesting strain-specific adaptation to enhanced phosphorus availability. In the ileum, *Candidatus arthromitus* (*p* < 0.001) and *Rombustia* (*p* < 0.05) showed a significant increase in relative abundance in PC compared to PF. Phytase supplementation reduced the relative abundance of *Lactobacillus* and *Streptococcus* (*p* < 0.05), while higher Ca concentration decreased that of *C. arthromitus* (*p* < 0.05). In the ceca, increases in the relative abundance of *Anaerostipes* (*p* < 0.05) and *Clostridia vadin BB60* were found for PC diets compared to PF (*p* < 0.001). The addition of phytase and Ca also significantly affected several genera, albeit the variations were less than 1 %.

Dietary PS, exogenous phytase, and Ca concentration modulated the gut microbiota, specifically influencing the abundance of key microorganisms like *Candidatus arthromitus, Anaerostipes,* and *Clostridia vadin BB60*, involved in phosphorus metabolism and overall broiler chickens' health.

## Introduction

The physical properties of poultry feed, particularly particle size (**PS**), can influence feed intake and performance of broiler chickens (Svihus et al., 2024a). The PS influences broiler chickens performance and feed preference (Svihus et al., 2024b), gizzard development and functionality (Svihus, 2011), and gut health (Itani et al., 2024). Feed processing techniques such as grinding and pelleting are energy-intensive, accounting for approximately 25 % and 40 % of total processing energy, respectively, underscoring the need to optimize PS not only for animal health but also for energy efficiency (Vukmirovic et al., 2010).

Studies have shown that particle coarse (**PC**) reduced digesta pH compared to those fed particle fine (**PF**) (Naderinejad et al., 2016; Svihus, 2011). This pH change can alter the physicochemical environment of the gastrointestinal tract (**GIT**), particularly affecting the efficacy of dietary enzymes such as phytase (Kumar et al., 2018). Moreover, variations in PS have been linked to shifts in gut microbiota composition, suggesting that feed structure may modulate microbial populations, their metabolic functions, and host nutrient metabolism (Ovi et al., 2021).

Phytases are phosphomonoesterases that initiate the dephosphorylation of phytate (Menezes-Blackburn et al., 2022), which is the primary storage form of phosphorus (**P**) in plant seeds such as cereal and legume grains (Mandha and Raes, 2023; Rodehutscord et al., 2022). Phytases mainly act in the upper sections of the GIT (Zeller et al., 2015), yielding less phosphorylated isomers and myo-inositol available for both host and microbial metabolic pathways (Sommerfeld et al., 2019; Wang et al., 2007). In broiler chickens, endogenous phytase expression is limited and subject to various dietary factors (Maenz and Classen, 1998).

Calcium carbonate (**CaCO_3_**) is the predominant calcium (**Ca**) supplement used in broiler chickens production worldwide (Kim et al., 2018). This compound is important in living organisms as both structural material and regulator of biological functions (Kolodkin-Gal et al., 2023). In broiler chickens, CaCO_3_ buffers the gut environment, which lowers the breakdown of inositol hexakisphosphate (**InsP₆**) before it reaches the intestine and reduces P digestibility (Bruggencate et al., 2004; Krieg et al., 2021). This is because of its high acid-binding capacity (Selle et al., 2009) and its tendency to form chelates that block enzymes from accessing phytate (Novotny et al., 2023). CaCO₃ may also affect the gut microbiota by promoting clumping of particles and biofilm formation (Xiong et al., 2022).

Feed particle size alters gut physiochemistry and nutrient utilization. Coarse particles enlarge and strengthen the gizzard, lowering its pH and that of the proximal small intestine. This acidification optimizes phytase activity (pH 4.0–5.5), enhancing phytate hydrolysis and release of myo-inositol and phosphorus (Wolfrum et al., 2024). Calcium carbonate solubility, and thus calcium absorption, is also improved at lower pH, while higher pH promotes poorly soluble calcium–phytate complexes (David et al., 2023). Consequently, coarse particle size indirectly enhances both phytase efficacy and calcium utilization by prolonging acidic retention in the upper gut, shaping the context for nutrient availability and microbial metabolism.

The chemical composition and physical structure of gut digesta and mucosa largely determine the microbial diversity and composition in the GIT of broiler chickens (Apajalahti et al., 2004). Predominant bacterial families such as *Lactobacillaceae, Lachnospiraceae, Oscillospiraceae,* and *Clostridiaceae* have been reported as the main colonizers of the GIT, with relative abundance variations depending on the GIT section (Borda-Molina et al., 2018; Moita et al., 2021) These bacteria exhibit a range of adaptation strategies, including acid and alkaline resistance (Li et al., 2021; Zhang et al., 2021) and specialized mechanisms for epithelial adherence (Hedblom et al., 2018).

This study aimed to investigate how dietary PS, exogenous phytase, and Ca concentration influence gut microbiota composition in broiler chickens. Specifically, we examined the core microbiota shared across feed and GIT samples, explored distribution patterns along the gut, and analyzed bacterial co-occurrence networks. We hypothesized that dietary changes would affect microbial composition, that a core microbiota would be consistently present across groups, and that bacterial genera would show distinct co-occurrence patterns within different GIT sections. If confirmed, this hypothesis would identify diet-responsive bacterial taxa and stable core members, providing mechanistic insight into diet–microbe interactions in the avian gut.

## Materials and methods

### Birds, housing and experimental diets

Samples were collected from the animal trial described by Wolfrum et al. (2024) that is briefly described here. The study was approved by the Regierungspräsidium Tübingen (approval No HOH 65/21_460a). Eight experimental diets were mixed as treatments arranged in a three-factorial design (2 × 2 × 2), by combining two levels of phytase (Natuphos E 5000 G, BASF SE, Ludwigshafen, Germany; 0, 1000 FTU/kg) with two Ca concentrations (4.9, 7.2 g/kg) and two PS (PF = 222 µm, and PC = 309 µm), see structure of experiment in Table 1. The diets were based on maize, soybean meal, rapeseed meal, and sunflower meal as detailed in Table 2, and formulated to be isocaloric and balanced for crude protein, metabolizable energy, and essential amino acids across particle size, phytase, and calcium treatments. The diets, meet or exceed the nutritional recommendations of Gesellschaft für Ernährungsphysiologie (GfE) (1999) guidelines. Titanium dioxide was incorporated as an indigestible marker with an inclusion level of 5 g/kg. To prevent selective feed intake, diets were pelleted. Analyzed nutrient profiles, physical and biochemical properties of the treatments as well as performance data, are reported in Wolfrum et al. (2024).

**Table 1:** Treatment structure of the experimental diets (phytase × calcium × particle size).

Diet	Phytase (FTU/kg)	Ca (g/kg)	Particle size (PS)
T1	0	4.9	PF (222 µm)
T2	0	7.2	PF (222 µm)
T3	1000	4.9	PF (222 µm)
T4	1000	7.2	PF (222 µm)
T5	0	4.9	PC (309 µm)
T6	0	7.2	PC (309 µm)
T7	1000	4.9	PC (309 µm)
T8	1000	7.2	PC (309 µm)

**Table 2:** Ingredient composition of the experimental diets fed from day 7 until slaughter (g/kg) (Wolfrum et al., 2024).

Calcium (g/kg)	4.9				7.2			
Particle size	coarse		fine		coarse		fine	
Phytase (FTU/kg)	0	1000	0	1000	0	1000	0	1000
Limestone^1^	4.9	4.9	4.9	4.9	10.5	10.5	10.5	10.5
Diatomaceous earth	5.6	5.6	5.6	5.6	0	0	0	0
Maize	561.7	561.7	561.7	561.7	561.7	561.7	561.7	561.7
Soybean meal	220	220	220	220	220	220	220	220
Rapeseed meal	75	75	75	75	75	75	75	75
Sunflower meal	75	75	75	75	75	75	75	75
Soybean oil	30	30	30	30	30	30	30	30
l-Lysine·HCl	0.6	0.6	0.6	0.6	0.6	0.6	0.6	0.6
dl-Methionine	1.8	1.8	1.8	1.8	1.8	1.8	1.8	1.8
l-Tryptophan	0.5	0.5	0.5	0.5	0.5	0.5	0.5	0.5
l-Valine	0.8	0.8	0.8	0.8	0.8	0.8	0.8	0.8
Trace element premix^2^	0.5	0.5	0.5	0.5	0.5	0.5	0.5	0.5
Vitamin premix^3^	2	2	2	2	2	2	2	2
Sodium bicarbonate	3	3	3	3	3	3	3	3
Sodium chloride	1	1	1	1	1	1	1	1
Choline chloride	2.6	2.6	2.6	2.6	2.6	2.6	2.6	2.6
Titanium dioxide	5	5	5	5	5	5	5	5
Calcium lignosulfonate	10	10	10	10	10	10	10	10

1 Contents of particles was declared as 10 % at 0.09 mm and 2 % at 0.18 mm.

2 Supplied per kg of diet: 25 mg calcium, 60 mg zinc, 25 mg iron, 80 mg manganese, 7.5 mg copper, 0.6 mg iodine, 0.2 mg selenium, 15 mg sepiolite.

3 Supplied per kg of diet: 0.6 g calcium, 10,000 IU vitamin A (retinyl acetate), 3,000 IU vitamin D_3_, 30 mg vitamin E (DL-α-tocopherol), 2.4 mg vitamin K_3_ (menadione), 100 µg biotin, 1.0 mg folic acid, 3.0 mg thiamine, 6.0 riboflavin, 6.0 mg pyridoxine, 30 µm cyanocobalamin, 50 mg niacin, 14 mg calcium d-pantothenate.

From day 0 to day 7, a cohort of 560 visually healthy, male Ross 308 broiler chickens hatchlings birds that were supplied by a commercial hatchery and randomly allocated into two groups; PF and PC; no pre-selection or screening of birds was conducted prior to allocation to avoid selection bias. Birds were housed in floor pens (3 × 4 m) with a litter of fresh wood shavings as bedding material. Both groups were initially fed starter diets, prepared in either PF or PC form. On day 7, groups of 10 birds were randomly assigned to the experimental treatments and allocated to 56 elevated metabolism units (1 m x 1 m x 1 m). The arrangement of treatments followed a complete randomized block design with seven repetitions for treatment. From days 7 to 22 and 23, the birds were fed the experimental diets. Feed and tap water were provided ad libitum throughout the experiment. The lighting schedule was set to 24 hours of light for the first three days, followed by 18 hours of light and 6 hours of darkness until the end of the experiment. The room temperature was gradually reduced from 34°C on day of hatch to 24°C by the end of the experiment. Birds were checked twice daily to monitor their health status.

### Sample collection

On day 7, samples of the experimental diets (**ED7**) were collected before filling the feeders. On days 22 and 23, birds were stunned with a gas mixture of 35 % CO_2_, 35 % N_2_, and 30 % O_2_, euthanized by CO_2_ exposure, and slaughtered. The gizzard, distal ileum (2.5 cm proximal to the ileocecal junction), and caeca from the 560 broiler chickens were longitudinally opened, and digesta was obtained with a sterile spoon and pooled on a metabolism unit basis. Samples from the experimental diets (**ED22**) were collected from the feeders on the slaughtering days. All collected samples were immediately stored at −80°C until analysis.

### DNA extraction, and sequencing library preparation

Microbial DNA was extracted from 280 samples using the FastDNA™SPIN Kit for Soil (MP Biomedical, Solon, OH, United States). Each extraction used 250 mg of material collected from the gizzard (*n* = 56), ileum (*n* = 56), ceca (*n* = 56), and two diets groups ED7 (*n* = 56) and ED22 (*n* = 56). DNA concentrations were measured using a NanoDrop 2000 Spectrophotometer (Thermo Fisher Scientific, Waltham, MA, United States) and stored at −20°C. Amplicon libraries targeting the V1-V2 region of the 16S rRNA gene were prepared using PrimeSTAR® HS DNA Polymerase kit (TaKaRa, Beijing, China), the region was chosen given the proven suitability for the taxonomic resolution requirements of broiler gut microbiota (Borda-Molina et al., 2020; Roth, 2023) following the protocol described by Kaewtapee et al. (Kaewtapee et al., 2017). Briefly, each of the three PCR steps was performed as follows: the first and second PCR reactions were carried out in 20 μL volumes containing 1 μL of template DNA, 0.2 μL of PrimeSTAR HS DNA polymerase, and 0.5 μL of each primer, forward primer CAAGRGTTHGATYMTGGCTCAG and the reverse primer TGCTGCCTCCCGTAGGAGT. The second PCR used 1 μL of the first PCR product as the template. The third PCR was performed in a 50 μL reaction volume using 1 μL of the second PCR product as the template. PCR cycling conditions included an initial denaturation at 95°C for 3 minutes, followed by 10 cycles for the first PCR, 15 cycles for the second, and 20 cycles for the third. Each cycle consisted of denaturation at 98°C for 10 seconds, annealing at 55°C for 10 seconds, and extension at 72°C for 45 seconds, with a final extension at 72°C for 2 minutes. PCR products were verified by agarose gel electrophoresis, purified, and normalized using the SequalPrep Normalization Kit (Invitrogen Inc., Carlsbad, CA, United States). Sequencing was performed on the Illumina NovaSeq 6000 platform, generating 250 bp paired-end reads.

### Bioinformatics and statistical analysis

Raw Fastq files were imported, demultiplexed, and processed using Qiime2 (v2022.8; (Bolyen et al., 2019)). Reads were trimmed using the q2-cutadapt plugin (Martin, 2011), and amplicon sequence variants (ASVs) were identified using the q2-dada2 plugin, which includes quality filtering, error correction, dereplication, and paired-end read merging (Callahan et al., 2016). Following common practice in 16S rRNA amplicon studies, sequences shorter than 200 bp or present in fewer than 50 samples were excluded from downstream analysis (Tan et al., 2020; Zhou et al., 2017), as those truncated and low-quality reads are unlikely to represent full-length target amplicons. Sklearn-based classifiers (Pedregosa et al., 2011) were generated with RESCRIPt (Robeson et al., 2021) using the Silva SSU-rRNA database (v.138.1, 16S 99 %; (Quast et al., 2013)). These were used for the taxonomic assignment of ASVs using VSEARCH (Rognes et al., 2016). Reads classified as mitochondrial, chloroplast, or unassigned were removed. Two samples were excluded due to low read counts. Alpha diversity (Shannon index) and beta diversity (Bray–Curtis dissimilarity) metrics were calculated using the q2‐diversity plugin following rarefaction. Principle Coordinate Analysis (PCoA) was used for data visualization.

Core microbiota members for each sample group were identified in QIIME 2 using the feature-table with the core-features command (Bolyen et al., 2019). A detection threshold of 0.01 % relative abundance and a minimum prevalence of 80 % across samples within each group were applied to define core ASVs. The resulting ASV lists were then compared across all groups to identify shared taxa. Venn diagrams illustrating these overlaps were generated using the “InteractiVenn” tool (Heberle et al., 2015). For this comparison, data were grouped solely by sample type, without accounting for experimental factors. To improve readability, ASVs were labeled using their assigned taxonomy along with the first four characters of their unique ASV identifier.

Statistical analysis of alpha-diversity across sample groups was conducted in R, version 4.3.1 (2023-10-16;(R Core Team, 2022)). The Shannon diversity index was calculated using the “vegan” package (Oksanen et al., 2024). To evaluate community composition differences, read counts were standardized per sample, and a Bray–Curtis dissimilarity matrix was generated. Differences in microbial community structure associated with experimental factors were assessed using permutational multivariate analysis of variance (PERMANOVA) via the “adonis” function in “vegan” package (Oksanen et al., 2024).

Data visualization was performed using the ggplot2 package in R to generate ordination plots, relative abundance bar charts, and diversity indices (Wickham, 2016). Prior to hypothesis testing, parametric assumptions, including normality, homogeneity of variances, and homoscedasticity, were evaluated, and depending on the results, either analysis of variance (ANOVA) using the “aov” function or non-parametric Kruskal–Wallis tests using the “kruskal.test” function were applied, as appropriate. Experimental factors significantly influencing response variables were further analyzed using a generalized linear model, implemented via the “glm” function in the general R package. The outputs of “glm” represent model-predicted estimates of the relative abundance used as the response variable.

The general model structure was defined as follows:

Response Variable=β₁ * Block + β₂ * PS + β₃ * Phytase + β₄ * Ca + β_5_ * PS:Phytase:Ca: *+ ε*

In this equation:

• Response Variable: Relative_Abundance or diversity indexes.
• Block: Denotes the Block factor (1-7).
• PS: The categorical variable denoting PS (PF or PC).
• Phytase: The numerical variable representing the amount of phytase 0, 1000 FTU/kg.
• Ca: The numerical variable representing the concentration of Ca (4.9, 7.2 g/kg)
• PS:Phytase:Ca: The interaction term between the factors PS, phytase, Ca.
• β₁, β₂, β₃, β4, and β5 represent the coefficients associated with the Block, PS, Phytase, Ca, and PS:Phytase:Ca, respectively.
• ε represents the error term.

While the model includes the three-way interaction term, statistical analysis revealed that main effects of individual factors (PS, phytase, Ca) were substantially more significant than interaction effects for the measured taxa, consistent with the relatively independent physiological mechanisms through which each factor modulates microbiota composition.

Bacterial genus co-occurrence patterns were analyzed using the differential gene correlation analysis R package (McKenzie et al., 2016). Samples were grouped according to the experimental factors: PS, Ca concentration, and phytase supplementation. Only moderate to strong correlations (*r* > 0.6) were considered for result interpretation. Correlations with *p*-values less than 0.05 were regarded as statistically significant.

## Results

### Sequencing overview

After quality control of the sequencing data, 278 samples were retained for downstream analysis. The dataset yielded an average of 24,975 ± 1,011 (mean ± standard error (**SEM**)) sequencing reads per sample. Reads were clustered into 6,330 ASVs assigned to 447 bacterial genera, with 11 genera showing an average relative abundance above 1 % across the dataset.

### Diversity indexes

**Fig. 1:**
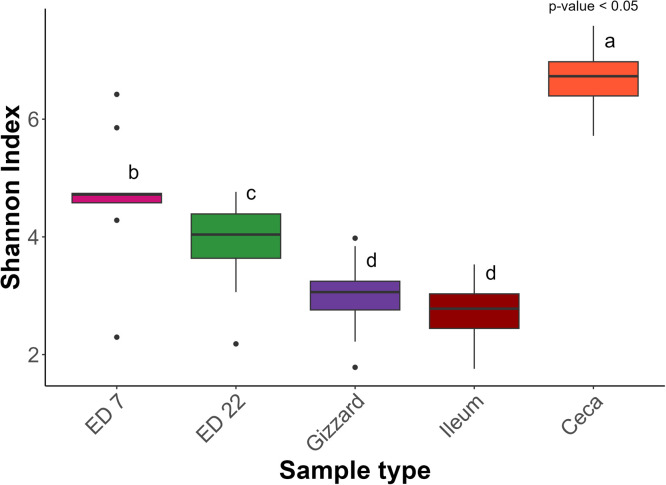
Boxplots showing Shannon diversity index values for samples from ED7, ED22, gizzard, ileum, and ceca. Different letters indicate statistically significant differences.

**Fig. 2:**
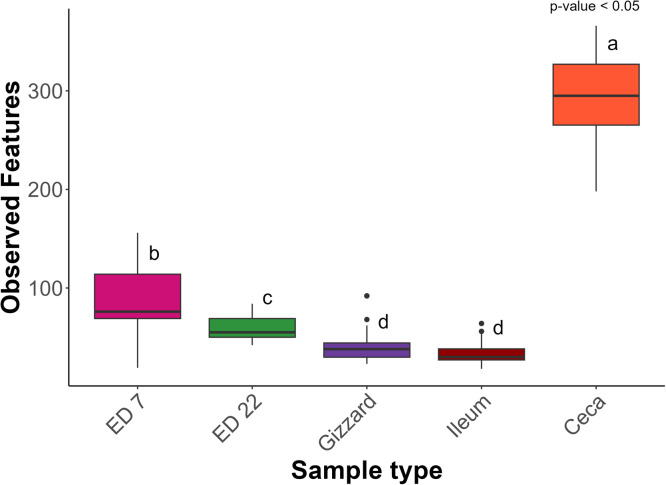
Boxplots showing Observed features values for samples from ED7, ED22, gizzard, ileum, and ceca. Different letters indicate statistically significant differences.

**Fig. 3:**
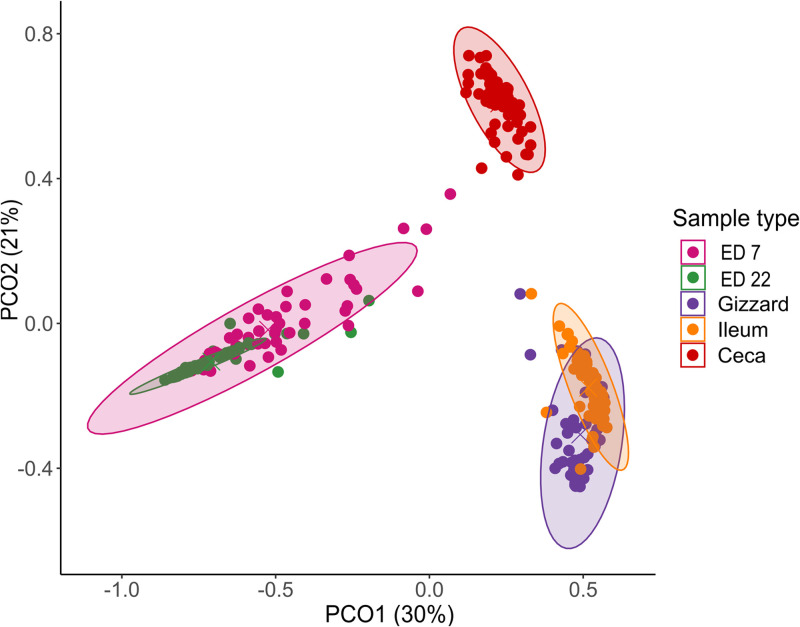
Principal Coordinates Analysis plot based on Bray–Curtis dissimilarity distances. Each point represents an individual sample, colored according to the sample type (ED7, ED22, gizzard, ileum, or ceca). Ellipses indicate 80 % confidence intervals around the group centroids.

**Fig. 4:**
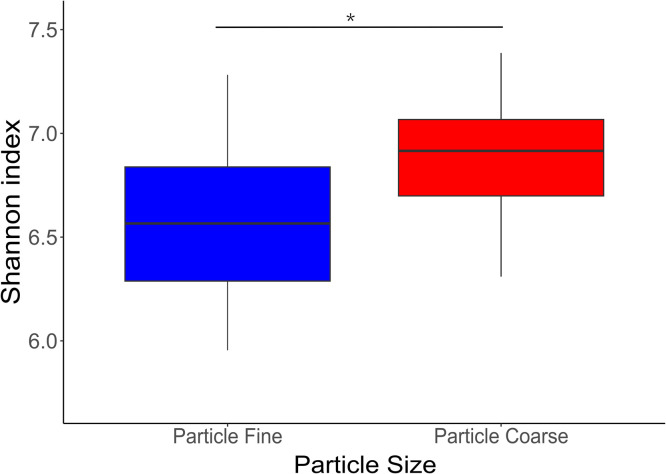
Boxplots of Shannon diversity index values for cecal samples grouped by particle size. Asterisk indicates a statistically significant difference (* *p* < 0.05).

**Fig. 5:**
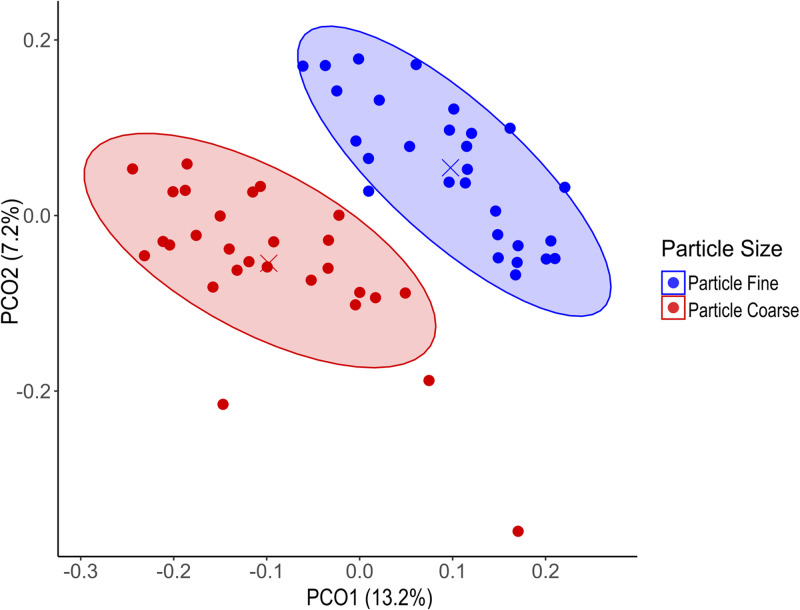
Principal Coordinates Analysis (PCoA) plot based on Bray–Curtis dissimilarity distances. Each point represents an individual sample, colored according to particle size treatment. Ellipses represent 80 % confidence intervals around the centroid of each particle size group.

An ANOVA indicated significant differences in alpha-diversity among the sample groups: gizzard, ileum, caeca, ED7, and ED22 (*p* < 0.05), based on the Shannon index and observed features. A Tukey post-hoc test showed no significant differences in the Shannon index (Fig. 1) or observed features (Fig. 2), between the gizzard and the ileum (*p* = 0.70), though both were significantly different from the other sample types (*p* < 0.001). Chao Index (Fig. S1) and Simpson Index (Fig. S2) showed no statistical differences across all the group of samples. Beta diversity, assessed using the Bray–Curtis dissimilarity index, also differed significantly among all sample groups, as determined by PERMANOVA (*p* < 0.05) (Fig. 3). In the gizzard and ileum, alpha diversity showed no significant differences between PF and PC treatments. In the ceca, alpha diversity was significantly lower in birds fed PF compared to those fed PC, as indicated by the Kruskal-Wallis test (*p* < 0.05) (Fig. 4), and beta-diversity was statistically different between the PF and PC diets (*p* < 0.05) (Fig. 5).

### Core microbiota assessment

**Fig. 6:**
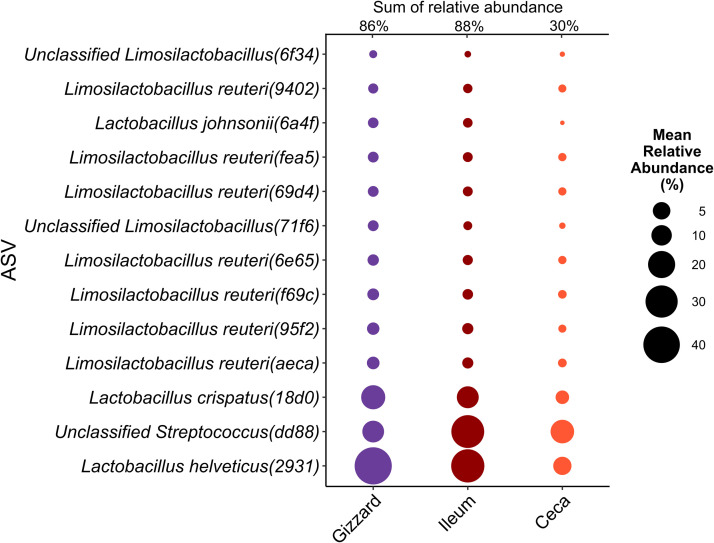
Bubble chart showing the mean relative abundance of shared ASVs across GIT sections. Bubble size corresponds to the relative abundance of each ASV in the respective section.

Thirteen ASVs were consistently detected across the gizzard, ileum, and ceca (Fig. S3). Among these, one ASV, unclassified *Streptococcus*(dd88), was shared across the ED7, gizzard, ileum, and ceca. Most of the shared ASVs found in the gizzard, ileum, and ceca were assigned to the genus *Limosilactobacillus*, contributing substantially to the microbial composition within each section: 86 % in the gizzard, 88 % in the ileum and 30 % in the ceca (Fig. 6). However, no single ASV was identified as core across all five sample groups, ED7, ED22, gizzard, ileum, and ceca.

**Fig. 7:**
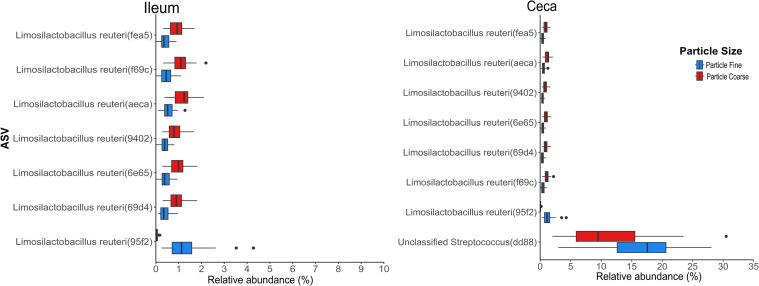
Relative abundance of persistent ASVs significantly affected by particle size in the ileum and ceca. Asterisks indicate statistical significance (*p-value<0.05).

Seven of the 13 ASVs showed significant variation in relative abundance due to PS (*p* < 0.05), as determined by a Kruskal-Wallis test. Specifically, *Limosilactobacillus reuteri(69d4), Limosilactobacillus reuteri(6e65), Limosilactobacillus reuteri(9402), Limosilactobacillus reuteri(aeca), Limosilactobacillus reuteri(f69c)*, and *Limosilactobacillus reuteri(fea5)* were more abundant in the PC diet, while *Limosilactobacillus reuteri(95f2)* was more prevalent in the PF diet (Fig. 6). In the ceca, a similar trend was observed, seven ASVs, including, *Limosilactobacillus reuteri(f69c), Limosilactobacillus reuteri(69d4), Limosilactobacillus reuteri(6e65), Limosilactobacillus reuteri(9402), Limosilactobacillus reuteri(aeca), Limosilactobacillus reuteri(fea5)*, were significantly more abundant in the PC diet (*p* < 0.05). Conversely, unclassified *Streptococcus(dd88)* and *Limosilactobacillus reuteri(95f2)* were more abundant in the PF diet (Fig. 7).

### Effects of experimental diets on GIT microbiota

The genera *Lactobacillus, Limosilactobacillus* and *Streptococcus* had a relative abundance higher than 1 % across the three studied GIT sections. *Lactobacillus* was the most abundant genus in the gizzard (60.9 % ±5.07) and the ileum (47.2 % ±5.34), while *Streptococcus* was the dominant genus in the ceca (14.1 % ±5.13).

In the gizzard, three genera collectively represented more than 95 % of the relative abundance across the dietary treatments: *Lactobacillus* (60.9 % ±5.07), *Limosilactobacillus* (23.6 % ±2.02), and *Streptococcus* (11.9 % ±4.81) (Fig. S4). No significant effects of dietary treatments were observed at genus level in this section.

In the ileum, five genera contributed more than 95 % of the relative abundance across treatments: *Lactobacillus* (47.2 % ±5.34), *Streptococcus* (31.5 % ±7.24), *Limosilactobacillus* (15.4 % ±2.85), *Candidatus Arthromitus* (3.66 % ±1.87), *Romboutsia* (1.12 % ±0.91) (Fig. S5). PC diets led to a significant increase in the relative abundance of *Candidatus arthromitus* (*p* < 0.001), *Limosilactobacillus* (*p* < 0.05), and *Romboutsia* (*p* < 0.05), compared to the PF diets. Phytase supplementation reduced the relative abundance of *Lactobacillus* (*p* < 0.05) and *Streptococcus* (*p* < 0.01), while higher Ca concentration decreased *Candidatus arthromitus* (*p* < 0.05) (Table 3).

**Table 3:** Generalized linear model (GLM) estimates showing the effects of particle size, phytase supplementation, and calcium concentration on the relative abundance of selected bacterial genera in the ileum. Values represent GLM model estimates of the relative abundance.

	Particle Fine		Particle Coarse		Phytase		Calcium	
*Candidatus arthromitus*	7.01	***	9.17	***	5.6 e-4		−0.69	*
*Lactobacillus*	35.60	***	36.98		−7.3 e-3	*	2.17	
*Limosilactobacillus*	9.12	*	8.66		−2.7 e-3		1.17	
*Romboutsia*	1.84		3.02	*	7.5 e-4		−0.25	
*Streptococcus*	47.5	***	43.32		8.9 e-3	*	−2.73	

GLM Model: Relative abundance ∼ particle size + phytase + Ca.

* *P* ≤ 0.05.

** *P* ≤ 0.01.

*** *P* ≤ 0.001.

The ceca had the greatest microbial diversity, with 19 genera with a relative abundance greater than 1 %. The most abundant taxa included *Streptococcus* (14.1 % ±5.13), *Lactobacillus* (12.4 % ±2.24), and *Clostridia vadin BB60* (9.96 % ±1.97) (Fig. S6). PC diets increased the relative abundance of *Anaerostipes* (*p* < 0.001) and *Clostridia vadin BB60* (*p* < 0.01), while decreasing *Negativibacillus* (*p* < 0.01), *Streptococcus* (*p* < 0.01), *Subdoligranulum* (*p* < 0.01), and *Ruminococcaceae UCG-005* (*p* < 0.01) compared to PF. Phytase supplementation significantly reduced the relative abundance of *Anaerostipes* (*p* < 0.001)*, Butyricicoccus* (*p* < 0.01)*, Lactobacillus* (*p* < 0.01)*, Negativibacillus* (*p* < 0.001)*, Sellimonas* (*p* < 0.01), and *Subdoligranulum* (*p* < 0.05), but increased that of *Streptococcus* (*p* < 0.001). Increased Ca concentrations were associated with reduced relative abundances of *Butyricicoccus* (*p* < 0.001)*, Faecalibacterium* (*p* < 0.001), *Lachnoclostridium* (*p* < 0.05), *Sellimonas* (*p* < 0.01), *Streptococcus* (*p* < 0.05), and *unclassified Lachospiraceae* (*p* < 0.01), and with elevated levels of *Limosilactobacillus* and *Ruminococcaceae UCG-005* (*p* < 0.05) (Table 4).

**Table 4:** Generalized linear model (GLM) estimates showing the effects of particle size, phytase supplementation, and calcium concentration on the relative abundance of selected bacterial genera in the ceca. Values represent GLM model estimates of the relative abundance.

	Particle Fine		Particle Coarse		Phytase		Calcium	
*Anaerostipes*	1.04	***	1.34	***	−5.1e-4	***	−2.5e-2	
*Butyricicoccus*	2.72	***	2.88		−2.7e-4	*	−0.21	***
*Clostridia vadinBB60*	4.77	*	7.30	**	−1.8 e-4		0.59	
*Faecalibacterium*	8.14	***	8.52		−7.4e-4		−0.53	***
*Lachnoclostridium*	1.29		1.38		−3.8e-5		−0.06	*
*Lactobacillus*	12.05	**	11.47		−3.5e-3	**	0.35	
*Limosilactobacillus*	1.53		2.10		−7.0e-4		0.76	**
*Negativibacillus*	2.59	***	2.19	**	−6.3e-4	***	−4.2e-2	
*Sellimonas*	1.42	***	1.39		−1.9e-4	**	−8.6e-2	**
*Streptococcus*	22.36	***	17.11	**	6.6e-3	***	−1.32	*
*Subdoligranulum*	3.34	***	2.79	**	−9.2e-4	*	−7.5e-2	
*Ruminococcaceae UCG-005*	−0.14		−0.78	**	7.6e-4	**	0.22	*
*Unclassified Lachnospiraceae*	14.44	***	15.32		−1.3e-4		−0.82	**

GLM Model: Relative abundance ∼ particle size + phytase + Ca.

* *P* ≤ 0.05.

** *P* ≤ 0.01.

*** *P* ≤ 0.001.

### Co-occurrence patterns across GIT

Significant positive correlations were identified between *Lactobacillus* and *Limosilactobacillus* in both the gizzard and the ileum, and these associations remained consistent regardless of dietary treatments (Table 5). Additional positive correlations were observed between *Clostridia UCG-014* and *Clostridia vadinBB60*, as well as between *Eisenbergiella* and *Erysipelatoclostridium*, specifically in the ileum and ceca. These correlations also persisted when samples were grouped according to experimental variables (Table 5). No correlations that spanned all GIT sections were detected.

**Table 5:** Pearson correlation coefficients and corresponding *p*-values for significant bacterial genus correlations consistently observed across sample groups.

	*Lactobacillus-Limosilactobacillus*			*Clostridia UCG 014-Clostridia vadinBB60*				*Eisenbergiella-Erysipelatoclostridium*		
	r		*p*-value	r		*p*-value	r			*p*-value
Gizzard										
Particle Coarse	0.89		***	0.96		***				
Particle Fine	0.67		***	0.92		***				
Phytase 0 (FTU/kg)	0.93		***	0.97		***				
Phytase 1000 (FTU/kg)	0.69		***	0.95		***				
Ca 5.5 (g/kg)	0.74		***	0.92		***				
Ca 8.0 (g/kg)	0.88		***	0.96		***				
Ileum										
Particle Coarse	0.96		***	0.96		***	0.64			***
Particle Fine	0.86		***	0.92		***	0.89			***
Phytase 0 (FTU/kg)	0.98		***	0.97		***	0.87			***
Phytase 1000 (FTU/kg)	0.81		***	0.95		***	0.86			***
Ca 5.5 (g/kg)	0.76		***	0.96		***	0.80			***
Ca 8.0 (g/kg)	0.97		***	0.92		***	0.91			***
Ceca										
Particle Coarse				0.80		***	0.82			***
Particle Fine				0.88		***	0.87			***
Phytase 0 (FTU/kg)				0.92		***	0.85			***
Phytase 1000 (FTU/kg)				0.93		***	0.82			***
Ca 5.5 (g/kg)				0.78		***	0.87			***
Ca 8.0 (g/kg)				0.88		***	0.80			***

*** *p*-value<0.001.

## Discussion

The present study investigated how dietary PS, phytase supplementation, and calcium addition modulate the GIT microbiota of broiler chickens. In the ileum, *Candidatus arthromitus* was significantly increased in broiler chickens fed PC diets, with no observed effect of phytase supplementation. This is consistent with enhanced prececal InsP_6_ disappearance under PC feeding in the absence of exogenous phytase (Wolfrum et al., 2024). *Candidatus arthromitus* has been associated with higher P utilization efficiency in Japanese quail (Borda-Molina et al., 2020; Vollmar et al., 2020), and its abundance has been shown to respond positively to increased dietary non-phytate P (Omotoso et al., 2023). Two physiological factors in this study likely promoted the observed increase of *Candidatus arthromitus*. First, PC feeding enhanced P availability in the small intestine (Wolfrum et al., 2024). This is relevant because *Candidatus arthromitus* possesses genes for the nonoxidative pentose phosphate pathway, which is essential for the synthesis of ribose-5-phosphate and other nucleotide sugars, both dependent on P as a structural component (Reiland, 2016; Reimer et al., 2021). Enzymes involved in this pathway, such as transketolase, also require thiamine pyrophosphate as a cofactor (Silverman, 2002), further linking intestinal P availability to microbial metabolic activity. Second, diets with PC have been associated with increased villus height in the small intestine (Novotný et al., 2023), potentially providing more surface area for microbial attachment. As a segmented filamentous bacterium, *Candidatus arthromitus* closely adheres to intestinal epithelial cells and contributes to mucosal immune development (Hedblom et al., 2018; Sanford, 1991). While we focused on digesta samples, enhanced intestinal morphology under PC feeding may have created more favorable conditions for *Candidatus arthromitus*. These mucosal effects likely extended to the digesta, explaining the increased relative abundance of this bacterium in our samples.

Thirteen ASVs were shared across the gizzard, ileum, and ceca, most belonging to *Limosilactobacillus*. Seven ASVs were significantly enriched in the ileum and eight in the ceca of broiler chickens fed PC. Previous work on the same birds has shown that PC reduced crop and gizzard pH (Wolfrum et al., 2024), likely favoring acid-tolerant bacteria such as *Limosilactobacillus*, which are well adapted to the broiler-chickens gut (Li et al., 2021; Rios-Galicia et al., 2024). Molecular factors linked to host gastric acid resistance have been identified as key contributors to host specialization and stable persistence of *Limosilactobacillus reuteri* in animal intestines (Li et al., 2021). Their persistence is further supported by metabolic traits, including glucose and galactose utilization (Reimer et al., 2021), and genes related to lactic acid production (Ksiezarek et al., 2022).

In the ceca, dietary interventions exerted distinct modulatory effects on microbial communities. the genus *Anaerostipes* increased when broiler chickens were fed PC. This genus has been previously identified in the cecal microbiota of broiler chickens (De Maesschalck et al., 2015; Eeckhaut et al., 2010). *Anaerostipes* species typically prefer a mildly acidic environment, with an optimum pH around 6.0 (Reimer et al., 2021). Isolates of *Anaerostipes* from human feces have demonstrated a strong capacity to convert lactate into butyrate, a short-chain fatty acid (SCFA) known for its beneficial effects on gut health (Duncan et al., 2004). In this study, birds fed PC diets also showed a higher abundance of lactic acid-producing bacteria in the ceca, primarily *Limosilactobacillus*, which likely contributed to increased lactate levels in the digesta. Elevated lactate concentrations may have provided a substrate for cross-feeding interactions, promoting the growth of lactate-utilizing genera such as *Anaerostipes*. This type of metabolic cross-feeding is documented in gut ecosystems and supports niche specialization among SCFA-producing bacteria. Furthermore, PC-fed broiler chickens exhibited elevated levels of myo-inositol in the terminal ileum (Wolfrum et al., 2024). Given that *Anaerostipes* can metabolize *myo-*inositol as a carbon source (Bui et al., 2021), it is plausible that unabsorbed myo-inositol entering the ceca contributed to its proliferation. The presence of *Anaerostipes* in the ceca is related to the host health, as a butyrate producer is a key energy source for colonocytes, enhances epithelial cell proliferation, reinforces the gut barrier, and exhibits anti-inflammatory properties (Eeckhaut et al., 2010).

The cecal bacteria belonging to *Clostridia vadin BB60* increased significantly in the broiler chickens fed with PC diets. This taxon exhibits a reproducible age-dependent colonization dynamic in cecal microbiota throughout broiler chickens development (Richards et al., 2019) and is often found among the dominant or highly abundant taxa in both the ileum and ceca (Choi and Kim, 2023; Richards et al., 2019; Ty et al., 2022). Despite its ecological relevance, the taxonomic identity of *Clostridia vadin BB60* remains poorly resolved below the class level (Parte et al., 2020; Quast et al., 2013), largely due to difficulties in cultivation (Crhanova et al., 2019; Richards et al., 2019), which has limited the knowledge about its metabolism and role in the microbiota (Hao et al., 2023). Recent studies, however, have begun to uncover potential roles for this group. *Clostridia vadin BB60* has been suggested a probiotic candidate in broiler chickens due to its negative correlation with inflammatory indicators, including serum IL-18, CXCL1, and CXCL2 levels (Liu et al., 2023). This anti-inflammatory potential may be linked to its positive correlation with straight-chain SCFA production (acetate, propionate, butyrate, valerate) and branched-chain SCFA (isobutyrate, isovalerate) (Cheng et al., 2021). Additionally, its increased abundance has been observed in ceca samples of broiler chickens following probiotic treatment after *Campylobacter jejuni* challenge (Ty et al., 2022). Although correlations with beneficial taxa have also been reported in mouse models (Feranmi, 2022), a negative association with n-butyric acid was observed in pigs (Sebastià et al., 2023), indicating that host species, diet, and microbial context may influence its function and impact.

In contrast, the genus *Streptococcus* showed a significant decrease in the ceca of PC fed broiler chickens. This observation aligns with previous findings where *Streptococcus* abundance declined when crimped kernels of maize silage were added to the diet (Ranjitkar et al., 2016). Conversely, other studies have reported increased *Streptococcus* levels when whole maize inclusion was elevated (Ovi et al., 2021) or when sieve sizes were expanded from 2 mm to 6 mm (Itani et al., 2024); however, these changes were not statistically significant. These inconsistencies suggest that *Streptococcus* populations may be sensitive only to substantial shifts in PS or specific feed physical properties rather than to moderate textural changes alone. The observed decrease of *Streptococcus* may also be attributed to microbial antagonism, particularly from *Limosilactobacillus*. We observed a significant increase in the vertically transmitted ASVs, which mainly belong to *Limosilactobacillus*. Previous in vitro research showed inhibitory effects of the *Limosilactobacillus* strains on *Streptococcus* during the first 18 h of co-culture (Nikawa et al., 2004); however, the exact mechanism of inhibition remains incompletely characterized.

The relative abundance of *Subdoligranulum* in the ceca was significantly reduced in broiler chickens fed PC diets. Cultured strains of this genus have been shown to produce butyrate (Hul et al., 2020; Wang et al., 2023). Given the concurrent increase in *Limosilactobacillus* abundance under PC feeding, one possible explanation for this reduction is competitive exclusion, whereby lactic acid bacteria outcompete other taxa for substrates or colonization niches. Environmental acidification provides another likely explanation. In laying hens, *Sudoligranulum* showed a significant decrease when the pH values decreased in the ileum (Jiang et al., 2023). The genome of *Subdoligranulum* encodes enzymes involved in adipate degradation (Reimer et al., 2021), a pathway related to the breakdown of acidity-regulating food additives (Kim et al., 2023). This suggests that *Subdoligranulum* may contribute to local pH buffering or modulation. Its decline under PC feeding may reflect both reduced substrate availability and a less favorable physicochemical environment.

Regarding phytase supplementation, in the ileum, *Lactobacillus* decreased, and *Streptococcus* increased when phytase was supplemented. This shift can be explained through sequential mechanisms. Phytase initiates the dephosphorylation of InsP_6_, which may be completed by endogenous phosphatases of either host or microbial origin (Vincent et al., 1992). Notably, *Streptococcus* contributes to this process through its intrinsic phosphatase activity (Reimer et al., 2021). Released myo-inositol serves as a carbon source for *Streptococcus* (Davis et al., 2022), potentially giving it a competitive advantage. Furthermore, *Streptococcus* can inhibit *Lactobacillus* via mutacin-type bacteriocins (Watanabe et al., 2021), further explaining the observed shifts.

In the ceca, phytase supplementation increased the relative abundance of *Streptococcus* and reduced the relative abundance of *Anaerostipes, Butyricicoccus, Lactobacillus, Negativibacillus, Sellimonas, Subdoligranulum*, and *Ruminococcaceae UCG-005*. It can be assumed that free *myo-*inositol increased after the stepwise degradation of InsP_6_, initiated by phytases and continued by endogenous phosphatases. Concurrently, the observed microbial shift likely reflects both Streptococcus's capacity to utilize myo-inositol as a carbon source (Davis et al., 2022). And its competitive advantage over butyrogenic genera (*Anaerostipes, Butyricicoccus*, etc.), mediated through bacteriocin production (Watanabe et al., 2021). Advantage particularly pronounced against butyrogenic genera that rely on cross-feeding for butyrate production via lactate or acetate metabolism, such as *Butyricicoccus* (Chang et al., 2020), *Anaerostipes*, and *Subdoligranulum* (Siptroth et al., 2023).

The addition of Ca further reduced the relative abundance of several genera in the ceca. This may reflect the role of Ca in promoting bacterial aggregation and altering spatial distribution within the gut (Macfarlane and Dillon, 2007). Ca is known as a non-bacterial agglutinin, capable of mediating microbial adhesion through charge neutralization and cation bridging mechanisms (Trunk et al., 2018). Specifically, Ca²⁺ ions can reduce electrostatic repulsion between negatively charged bacterial surfaces, promoting aggregation and enhancing adherence to mucus or epithelial surfaces (Xiong et al., 2022). Such interactions may shift bacteria from the digesta to mucosa-associated niches, altering their relative abundance in luminal samples.

Regarding vertical transmission, Unclassified *Streptococcus* (dd88) was detected in ED7, gizzard, ileum, and ceca samples, surpassing the ASV detection threshold of 80 %. This contrasts with our previous study (Rubio-Cervantes et al., 2025), where no vertical transmission from feed to the GIT was observed, as *Unclassified Streptococcus* (dd88) did not meet the detection threshold. These differences may reflect microbial adaptation to feed and GIT conditions. The intestinal microbiota of broiler chickens is known to be highly dependent on the surrounding environment (Ding et al., 2017). We speculate that in the current experiment, this ASV found favorable environmental conditions in both ED7 and the GIT, which were absent in the earlier trial (Rubio-Cervantes et al., 2025).

Bacterial co-occurrences were inferred from treatment-specific Pearson correlation matrices computed separately for each GIT section (gizzard, ileum, ceca). The co-occurrence analysis revealed a strong and consistent positive association between *Lactobacillus* and *Limosilactobacillus* across the gizzard and ileum. This mutual relationship may be explained by shared ecological and physiological traits, including acid tolerance and exopolysaccharides production (Zheng et al., 2020). Exopolysaccharides support biofilm formation and exhibit antioxidant, immunomodulatory, and anti-inflammatory properties (Tarannum et al., 2024), which may facilitate cooperative colonization and resilience in the gut, independent of the tested dietary variations. In contrast, the correlation between *Eisenbergiella* and *Erysipelatoclostridium*, both genera linked to pro-inflammatory signatures, was significantly reduced in broiler chickens fed PC diets. Similar results have been previously reported in humans (Qin et al., 2024), and associated with inflammatory processes (Ashraf et al., 2024). The concurrent increase in the *Lactobacillus-Limosilactobacillus* co-occurrence and the weakening of the *Eisenbergiella-Erysipelatoclostridium* association in PC-fed broiler chickens highlights the potential for dietary structure to selectively modulate microbial interactions. These findings suggest that diets with PC properties may promote probiotic partnerships while disrupting potentially pro-inflammatory microbial networks, thereby influencing the gut ecosystem beyond changes in individual taxa.

## Conclusions and applications

These findings suggest that PS of the feed can be strategically used to enrich beneficial gut bacteria associated with higher nutrient utilization. Significant differences in microbial abundance, composition, and inter-genus correlations were observed in response to PS, suggesting that physical properties of feed modulate not only individual taxa but also microbial interactions throughout the GIT tract.

Exogenous phytase influenced cecal microbiota composition, likely through its effects on phosphate and myo-inositol availability in the upper GIT. Meanwhile, Ca supplementation altered microbial profiles, potentially due to its function as a bacterial agglutinant, promoting redistribution of microbes toward mucosal surfaces.

Within the range tested, feeding broiler chickens with PC diets increased the abundance of bacterial taxa associated with probiotic activity and P utilization, such as *Clostridia vadin BB60* and *Candidatus Arthromitus*, which indicates that physical feed structure is an important modulator of the gut microbiota. Given that *Candidatus Arthromitus* is not fully described, our findings also contribute new insights into its distribution and potential ecological role in the broiler ileum.

## Funding

This work was partly funded by 10.13039/501100001659Deutsche Forschungsgemeinschaft (DFG, German Research Foundation) – 328017493/GRK 2366 (Sino-German International Research Training Group AMAIZE-P).

## Data availability

The data sets generated and/or analyzed during the current study are available in the PRJEB82764 repository.

## CRediT authorship contribution statement

**Ismael Rubio-Cervantes:** Writing – review & editing, Writing – original draft, Visualization, Methodology, Investigation, Formal analysis. **Stephanie Wolfrum:** Writing – review & editing, Methodology. **Wolfgang Siegert:** Writing – review & editing, Methodology. **Markus Rodehutscord:** Writing – review & editing, Supervision, Conceptualization. **Amélia Camarinha-Silva:** Writing – review & editing, Supervision, Methodology, Funding acquisition, Conceptualization.

## Disclosures

The authors declare the following financial interests/personal relationships which may be considered as potential competing interests:

Ismael Rubio-Cervantes reports financial support was provided by Deutsche Forschungsgemeinschaft. If there are other authors, they declare that they have no known competing financial interests or personal relationships that could have appeared to influence the work reported in this paper.

## Appendix Supplementary materials

Image, application 1

## Footnotes

Supplementary material associated with this article can be found, in the online version, at doi:10.1016/j.psj.2025.106209.
