# Supplementary material for: Microbiota responses to feed particle size, calcium concentration, and phytase supplementation in broiler chickens
Source: Poult Sci. 2025 Dec 6;105(1):106209. doi: 10.1016/j.psj.2025.106209 (PMC12752539; doi:10.1016/j.psj.2025.106209)
Supplement: Supplementary file 1 [file mmc1.pdf]

MICROBIOTA RESPONSES TO FEED PARTICLE SIZE, CALCIUM CONCENTRATION, AND PHYTASE SUPPLEMENTATION IN BROILER CHICKENS

Ismael Rubio-Cervantes[*], Stephanie Wolfrum[*], Wolfgang Siegert[†], Markus Rodehutscord[*], Amelia Camarinha-Silva[*,1]

[*]Institute of Animal Science, University of Hohenheim, 70599, Stuttgart, Germany

[†]Department of Animal Sciences, University of Göttingen, 37077 Göttingen, Germany

[1]Corresponding author: Prof. Dr. Amélia Camarinha Silva

amelia.silva@uni-hohenheim.de, +49 711 459 23064

Emil-Wolff-Str. 10, 70599 Stuttgart, Germany

Scientific section: Metabolism and Nutrition

SUPPLEMENTARY FIGURES

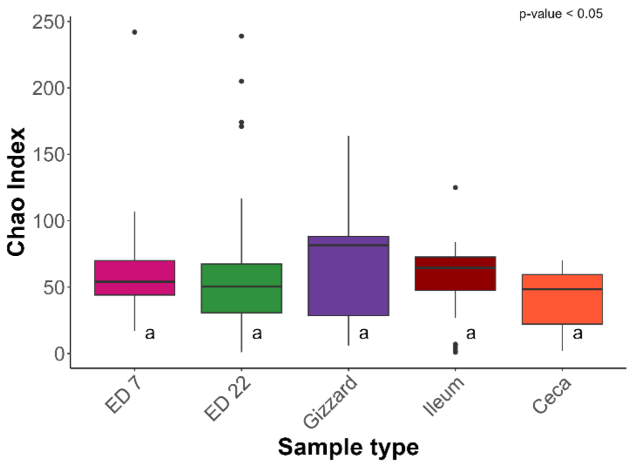

Fig. S1.- Boxplots showing Chao diversity index values for samples from ED7, ED22, gizzard, ileum, and ceca. Different letters indicate statistically significant differences.

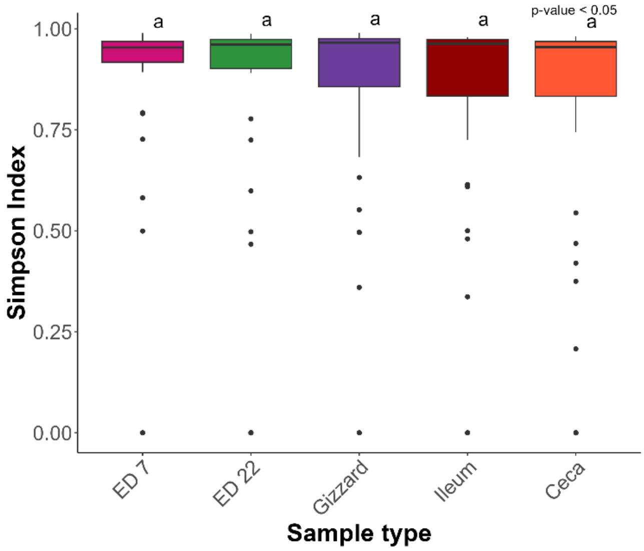

Fig. S2.- Boxplots showing Simpson diversity index values for samples from ED7, ED22, gizzard, ileum, and ceca. Different letters indicate statistically significant differences.

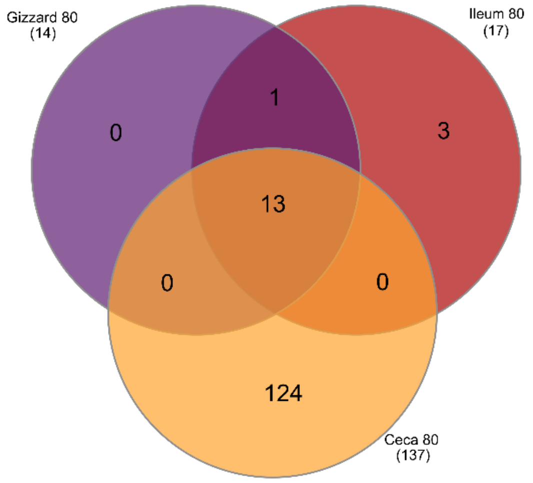

Fig. S3.- Venn diagram showing the distribution of total ASVs across GIT sections. Numbers in parentheses indicate the total observed ASVs in each section. Core members were defined using a detection threshold of 0.01% relative abundance and a minimum prevalence of 80% across samples.

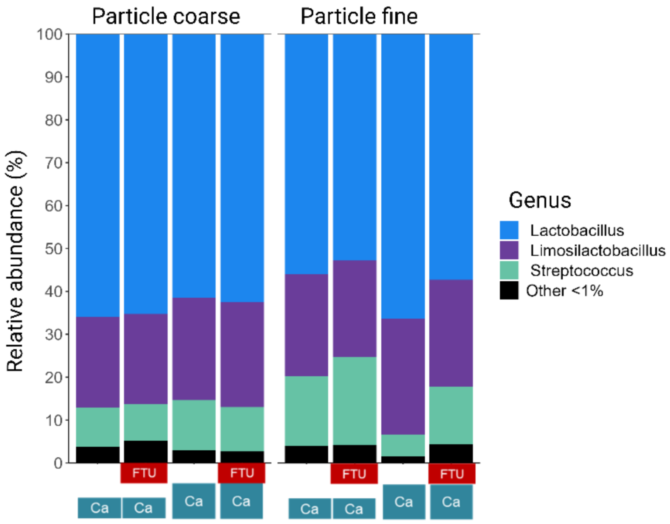

Fig. S4.- Barplots showing microbiota composition at the genus level in the gizzard across different combinations of particle size, calcium (Ca) concentration, and phytase (FTU) supplementation.

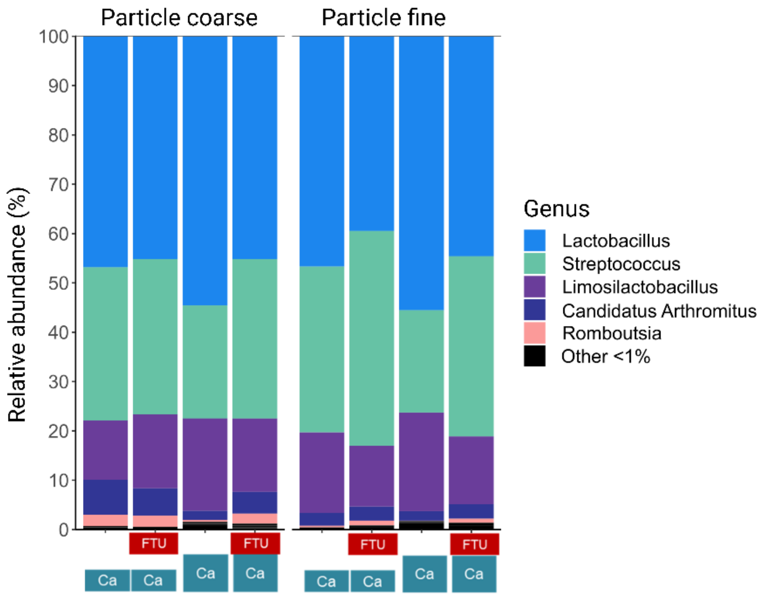

Fig. S5.- Barplots showing microbiota composition at the genus level in the ileum across different combinations of particle size, calcium (Ca) concentration, and phytase (FTU) supplementation.

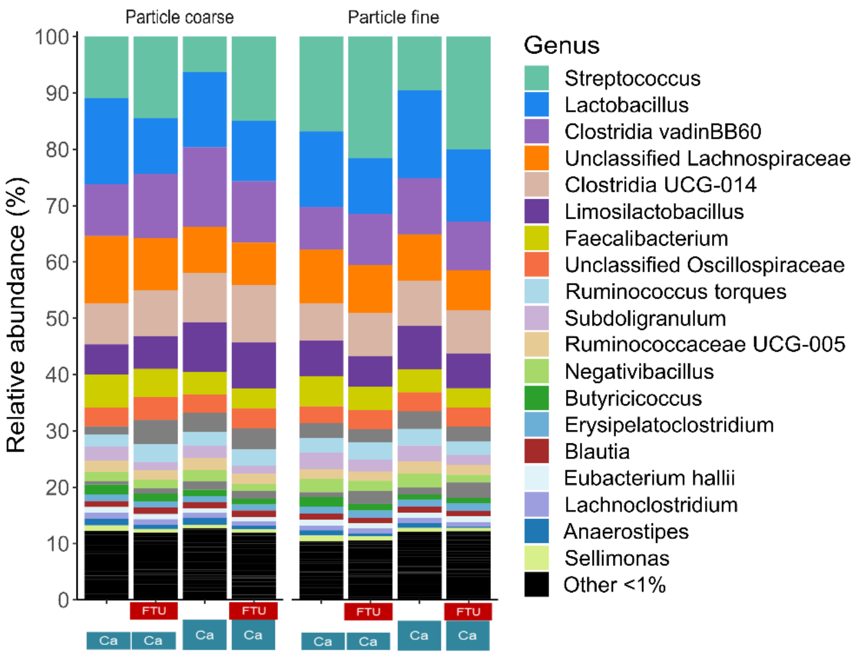

Fig. S6.- Barplots showing microbiota composition at the genus level in the ceca across different combinations of particle size, calcium (Ca) concentration, and phytase (FTU) supplementation.
